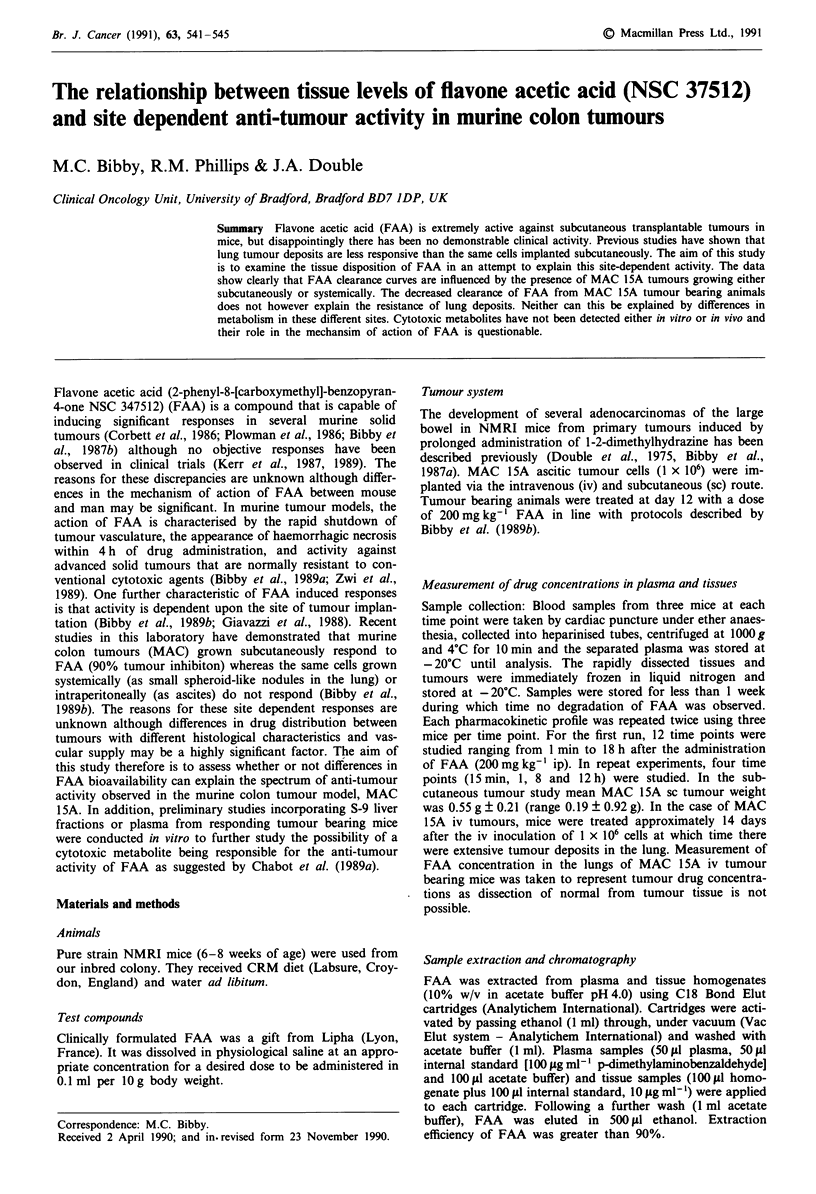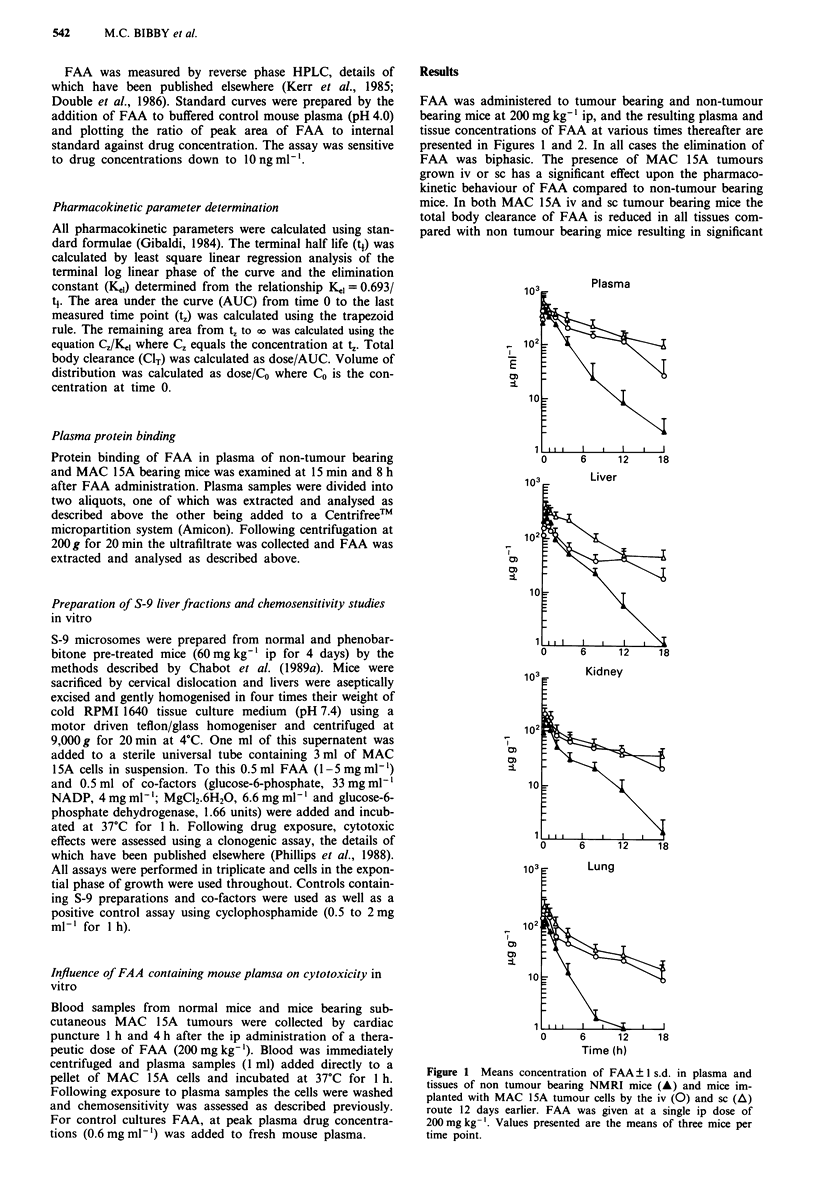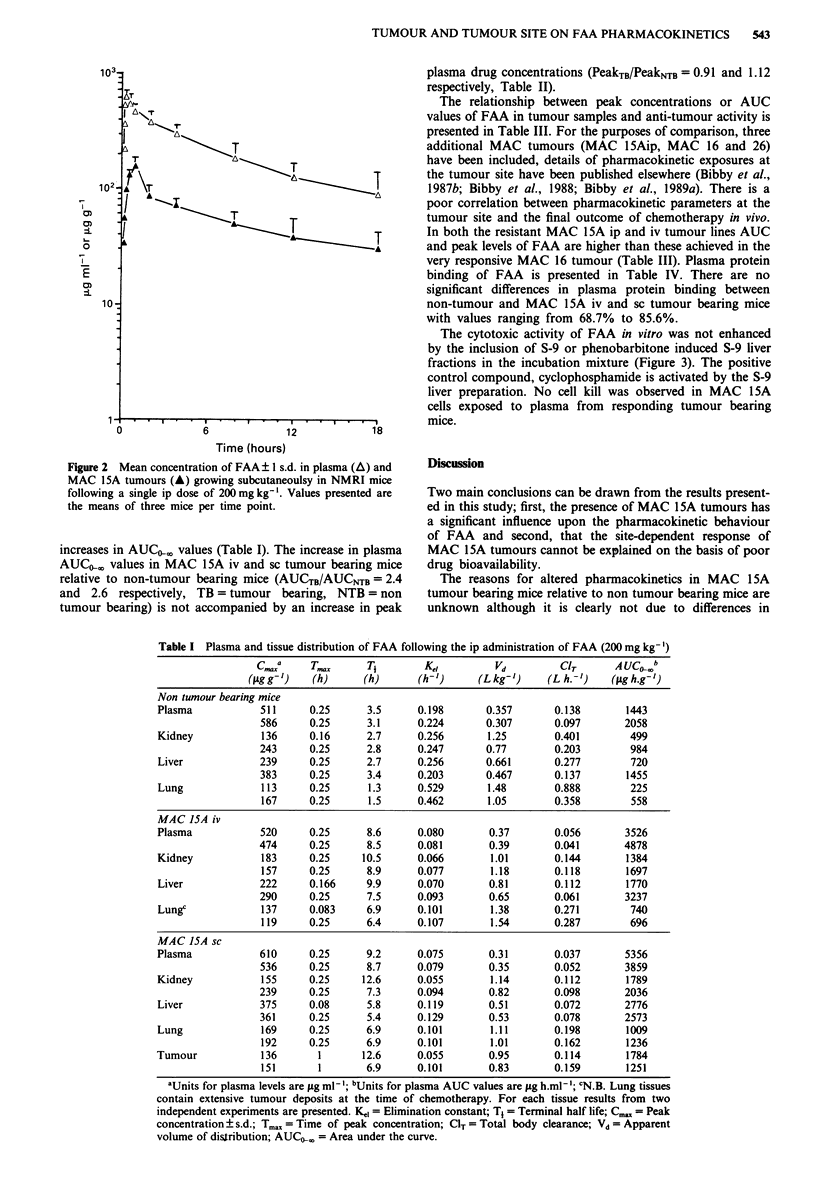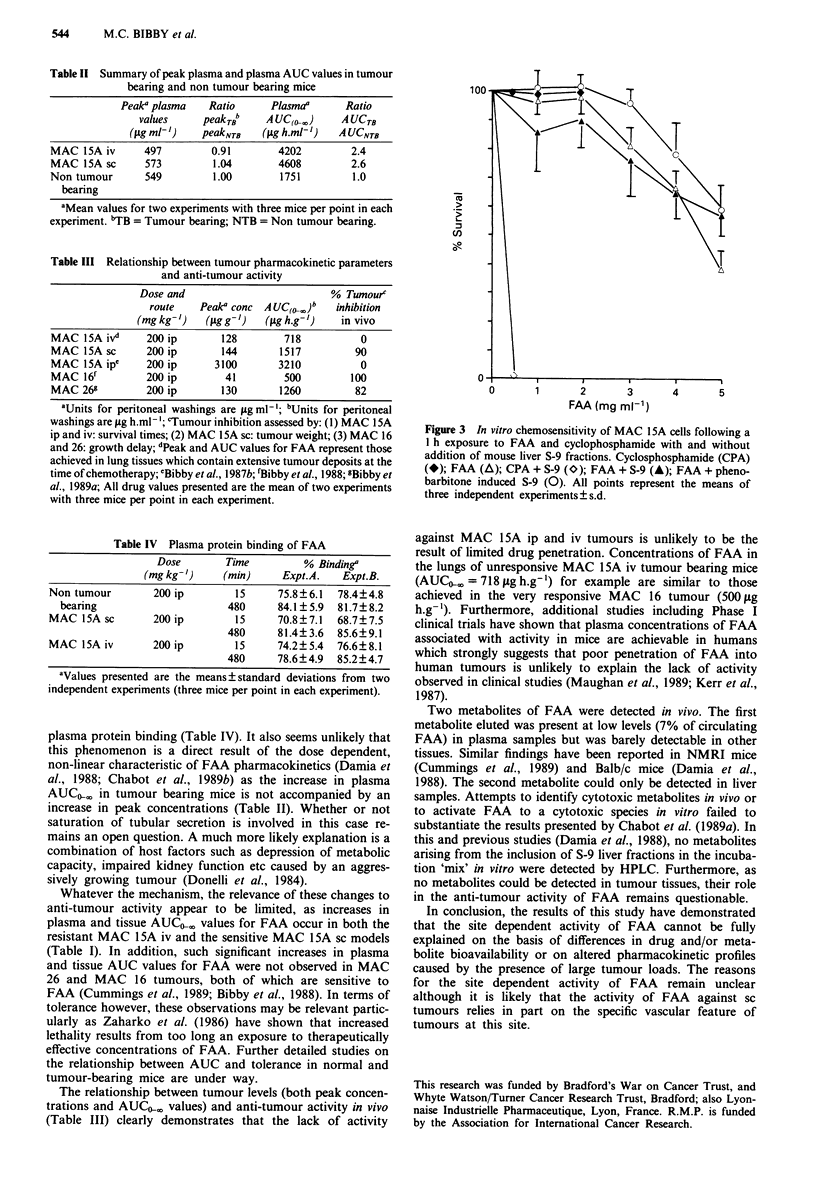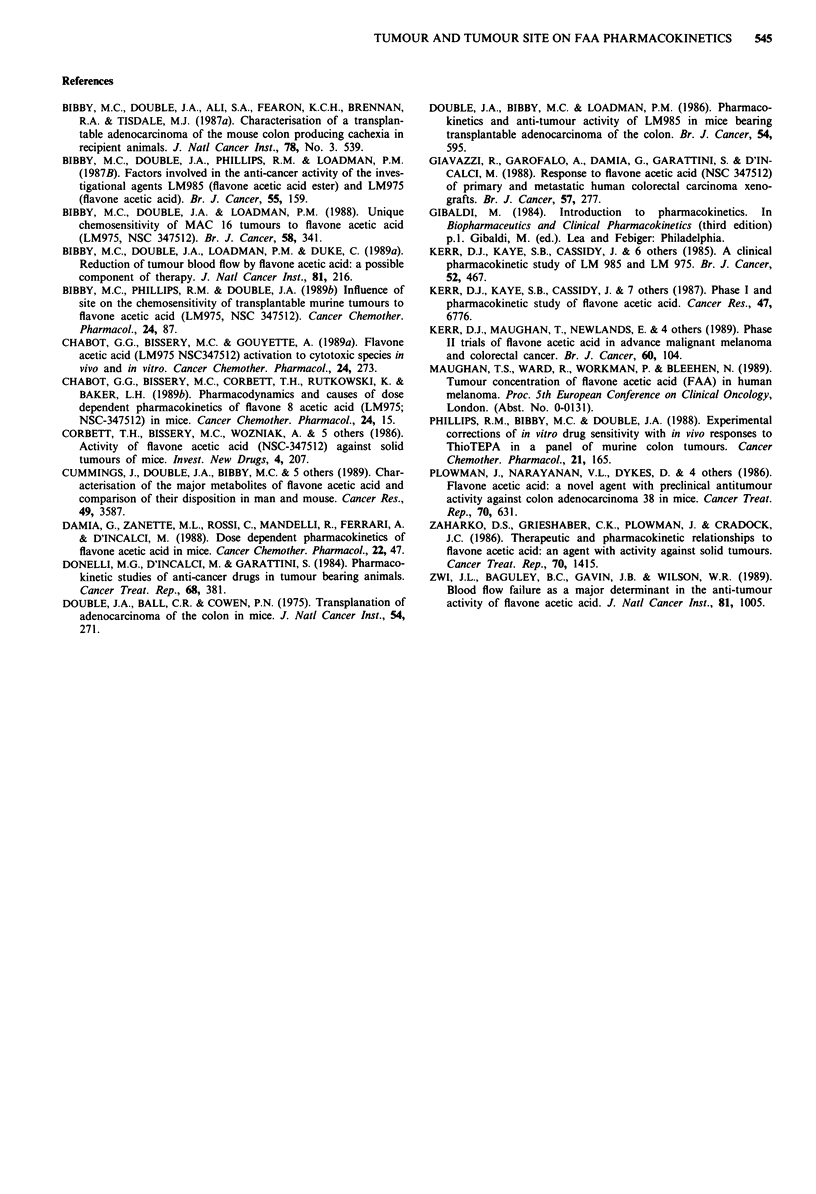# The relationship between tissue levels of flavone acetic acid (NSC 347512) and site dependent anti-tumour activity in murine colon tumours.

**DOI:** 10.1038/bjc.1991.127

**Published:** 1991-04

**Authors:** M. C. Bibby, R. M. Phillips, J. A. Double

**Affiliations:** Clinical Oncology Unit, University of Bradford, UK.

## Abstract

Flavone acetic acid (FAA) is extremely active against subcutaneous transplantable tumours in mice, but disappointingly there has been no demonstrable clinical activity. Previous studies have shown that lung tumour deposits are less responsive than the same cells implanted subcutaneously. The aim of this study is to examine the tissue disposition of FAA in an attempt to explain this site-dependent activity. The data show clearly that FAA clearance curves are influenced by the presence of MAC 15A tumours growing either subcutaneously or systemically. The decreased clearance of FAA from MAC 15A tumour bearing animals does not however explain the resistance of lung deposits. Neither can this be explained by differences in metabolism in these different sites. Cytotoxic metabolites have not been detected either in vitro or in vivo and their role in the mechanism of action of FAA is questionable.


Br. J. Cancer (1991), 63, 541–545 © Macmillan Press Ltd., 1991

### The relationship between tissue levels of flavone acetic acid (NSC 37512) and site dependent anti-tumour activity in murine colon tumours

M.C. Bibby, R.M. Phillips & J.A. Double

*Clinical Oncology Unit, University of Bradford, Bradford BD7 1DP, UK*

**Summary** Flavone acetic acid (FAA) is extremely active against subcutaneous transplantable tumours in mice, but disappointingly there has been no demonstrable clinical activity. Previous studies have shown that lung tumour deposits are less responsive than the same cells implanted subcutaneously. The aim of this study is to examine the tissue disposition of FAA in an attempt to explain this site-dependent activity. The data show clearly that FAA clearance curves are influenced by the presence of MAC 15A tumours growing either subcutaneously or systemically. The decreased clearance of FAA from MAC 15A tumour bearing animals does not however explain the resistance of lung deposits. Neither can this be explained by differences in metabolism in these different sites. Cytotoxic metabolites have not been detected either *in vitro* or *in vivo* and their role in the mechansim of action of FAA is questionable.

Flavone acetic acid (2-phenyl-8-[carboxymethyl]-benzopyran-4-one NSC 347512) (FAA) is a compound that is capable of inducing significant responses in several murine solid tumours (Corbett *et al.*, 1986; Plowman *et al.*, 1986; Bibby *et al.*, 1987*b*) although no objective responses have been observed in clinical trials (Kerr *et al.*, 1987, 1989). The reasons for these discrepancies are unknown although differences in the mechanism of action of FAA between mouse and man may be significant. In murine tumour models, the action of FAA is characterised by the rapid shutdown of tumour vasculature, the appearance of haemorrhagic necrosis within 4 h of drug administration, and activity against advanced solid tumours that are normally resistant to conventional cytotoxic agents (Bibby *et al.*, 1989*a*; Zwi *et al.*, 1989). One further characteristic of FAA induced responses is that activity is dependent upon the site of tumour implantation (Bibby *et al.*, 1989*b*; Giavazzi *et al.*, 1988). Recent studies in this laboratory have demonstrated that murine colon tumours (MAC) grown subcutaneously respond to FAA (90% tumour inhibiton) whereas the same cells grown systemically (as small spheroid-like nodules in the lung) or intraperitoneally (as ascites) do not respond (Bibby *et al.*, 1989*b*). The reasons for these site dependent responses are unknown although differences in drug distribution between tumours with different histological characteristics and vascular supply may be a highly significant factor. The aim of this study therefore is to assess whether or not differences in FAA bioavailability can explain the spectrum of anti-tumour activity observed in the murine colon tumour model, MAC 15A. In addition, preliminary studies incorporating S-9 liver fractions or plasma from responding tumour bearing mice were conducted *in vitro* to further study the possibility of a cytotoxic metabolite being responsible for the anti-tumour activity of FAA as suggested by Chabot *et al.* (1989*a*).

### Materials and methods

### *Animals*

Pure strain NMRI mice (6–8 weeks of age) were used from our inbred colony. They received CRM diet (Labsure, Croydon, England) and water *ad libitum*.

### *Test compounds*

Clinically formulated FAA was a gift from Lipha (Lyon, France). It was dissolved in physiological saline at an appropriate concentration for a desired dose to be administered in 0.1 ml per 10 g body weight.

Correspondence: M.C. Bibby.
Received 2 April 1990; and in revised form 23 November 1990.

### *Tumour system*

The development of several adenocarcinomas of the large bowel in NMRI mice from primary tumours induced by prolonged administration of 1-2-dimethylhydrazine has been described previously (Double *et al.*, 1975, Bibby *et al.*, 1987*a*). MAC 15A ascitic tumour cells ($1 \times 10^6$) were implanted via the intravenous (iv) and subcutaneous (sc) route. Tumour bearing animals were treated at day 12 with a dose of 200 mg kg$^{-1}$ FAA in line with protocols described by Bibby *et al.* (1989*b*).

### *Measurement of drug concentrations in plasma and tissues*

Sample collection: Blood samples from three mice at each time point were taken by cardiac puncture under ether anaesthesia, collected into heparinised tubes, centrifuged at 1000 *g* and 4°C for 10 min and the separated plasma was stored at −20°C until analysis. The rapidly dissected tissues and tumours were immediately frozen in liquid nitrogen and stored at −20°C. Samples were stored for less than 1 week during which time no degradation of FAA was observed. Each pharmacokinetic profile was repeated twice using three mice per time point. For the first run, 12 time points were studied ranging from 1 min to 18 h after the administration of FAA (200 mg kg$^{-1}$ ip). In repeat experiments, four time points (15 min, 1, 8 and 12 h) were studied. In the subcutaneous tumour study mean MAC 15A sc tumour weight was 0.55 g ± 0.21 (range 0.19 ± 0.92 g). In the case of MAC 15A iv tumours, mice were treated approximately 14 days after the iv inoculation of $1 \times 10^6$ cells at which time there were extensive tumour deposits in the lung. Measurement of FAA concentration in the lungs of MAC 15A iv tumour bearing mice was taken to represent tumour drug concentrations as dissection of normal from tumour tissue is not possible.

### *Sample extraction and chromatography*

FAA was extracted from plasma and tissue homogenates (10% w/v in acetate buffer pH 4.0) using C18 Bond Elut cartridges (Analytichem International). Cartridges were activated by passing ethanol (1 ml) through, under vacuum (Vac Elut system – Analytichem International) and washed with acetate buffer (1 ml). Plasma samples (50 µl plasma, 50 µl internal standard [100 µg ml$^{-1}$ p-dimethylaminobenzaldehyde] and 100 µl acetate buffer) and tissue samples (100 µl homogenate plus 100 µl internal standard, 10 µg ml$^{-1}$) were applied to each cartridge. Following a further wash (1 ml acetate buffer), FAA was eluted in 500 µl ethanol. Extraction efficiency of FAA was greater than 90%.

FAA was measured by reverse phase HPLC, details of which have been published elsewhere (Kerr et al., 1985; Double et al., 1986). Standard curves were prepared by the addition of FAA to buffered control mouse plasma (pH 4.0) and plotting the ratio of peak area of FAA to internal standard against drug concentration. The assay was sensitive to drug concentrations down to $10\,ng\,ml^{-1}$.

*Pharmacokinetic parameter determination*

All pharmacokinetic parameters were calculated using standard formulae (Gibaldi, 1984). The terminal half life ($t_{\frac{1}{2}}$) was calculated by least square linear regression analysis of the terminal log linear phase of the curve and the elimination constant ($K_{el}$) determined from the relationship $K_{el} = 0.693/t_{\frac{1}{2}}$. The area under the curve (AUC) from time 0 to the last measured time point ($t_z$) was calculated using the trapezoid rule. The remaining area from $t_z$ to $\infty$ was calculated using the equation $C_z/K_{el}$ where $C_z$ equals the concentration at $t_z$. Total body clearance ($Cl_T$) was calculated as dose/AUC. Volume of distribution was calculated as dose/$C_0$ where $C_0$ is the concentration at time 0.

*Plasma protein binding*

Protein binding of FAA in plasma of non-tumour bearing and MAC 15A bearing mice was examined at 15 min and 8 h after FAA administration. Plasma samples were divided into two aliquots, one of which was extracted and analysed as described above the other being added to a Centrifree™ micropartition system (Amicon). Following centrifugation at 200 *g* for 20 min the ultrafiltrate was collected and FAA was extracted and analysed as described above.

*Preparation of S-9 liver fractions and chemosensitivity studies* in vitro

S-9 microsomes were prepared from normal and phenobarbitone pre-treated mice ($60\,mg\,kg^{-1}$ ip for 4 days) by the methods described by Chabot et al. (1989*a*). Mice were sacrificed by cervical dislocation and livers were aseptically excised and gently homogenised in four times their weight of cold RPMI 1640 tissue culture medium (pH 7.4) using a motor driven teflon/glass homogeniser and centrifuged at 9,000 *g* for 20 min at 4°C. One ml of this supernatent was added to a sterile universal tube containing 3 ml of MAC 15A cells in suspension. To this 0.5 ml FAA ($1-5\,mg\,ml^{-1}$) and 0.5 ml of co-factors (glucose-6-phosphate, $33\,mg\,ml^{-1}$ NADP, $4\,mg\,ml^{-1}$; $MgCl_2.6H_2O$, $6.6\,mg\,ml^{-1}$ and glucose-6-phosphate dehydrogenase, 1.66 units) were added and incubated at 37°C for 1 h. Following drug exposure, cytotoxic effects were assessed using a clonogenic assay, the details of which have been published elsewhere (Phillips et al., 1988). All assays were performed in triplicate and cells in the exponential phase of growth were used throughout. Controls containing S-9 preparations and co-factors were used as well as a positive control assay using cyclophosphamide (0.5 to 2 mg $ml^{-1}$ for 1 h).

*Influence of FAA containing mouse plamsa on cytotoxicity* in vitro

Blood samples from normal mice and mice bearing subcutaneous MAC 15A tumours were collected by cardiac puncture 1 h and 4 h after the ip administration of a therapeutic dose of FAA ($200\,mg\,kg^{-1}$). Blood was immediately centrifuged and plasma samples (1 ml) added directly to a pellet of MAC 15A cells and incubated at 37°C for 1 h. Following exposure to plasma samples the cells were washed and chemosensitivity was assessed as described previously. For control cultures FAA, at peak plasma drug concentrations ($0.6\,mg\,ml^{-1}$) was added to fresh mouse plasma.

### Results

FAA was administered to tumour bearing and non-tumour bearing mice at $200\,mg\,kg^{-1}$ ip, and the resulting plasma and tissue concentrations of FAA at various times thereafter are presented in Figures 1 and 2. In all cases the elimination of FAA was biphasic. The presence of MAC 15A tumours grown iv or sc has a significant effect upon the pharmacokinetic behaviour of FAA compared to non-tumour bearing mice. In both MAC 15A iv and sc tumour bearing mice the total body clearance of FAA is reduced in all tissues compared with non tumour bearing mice resulting in significant

**Figure 1** Means concentration of FAA ± 1 s.d. in plasma and tissues of non tumour bearing NMRI mice (▲) and mice implanted with MAC 15A tumour cells by the iv (O) and sc (Δ) route 12 days earlier. FAA was given at a single ip dose of $200\,mg\,kg^{-1}$. Values presented are the means of three mice per time point.

**Figure 2** Mean concentration of FAA ± 1 s.d. in plasma (Δ) and MAC 15A tumours (▲) growing subcutaneoulsy in NMRI mice following a single ip dose of 200 mg kg$^{-1}$. Values presented are the means of three mice per time point.

increases in $AUC_{0-\infty}$ values (Table I). The increase in plasma $AUC_{0-\infty}$ values in MAC 15A iv and sc tumour bearing mice relative to non-tumour bearing mice ($AUC_{TB}/AUC_{NTB} = 2.4$ and 2.6 respectively, TB = tumour bearing, NTB = non tumour bearing) is not accompanied by an increase in peak plasma drug concentrations ($Peak_{TB}/Peak_{NTB} = 0.91$ and 1.12 respectively, Table II).

The relationship between peak concentrations or AUC values of FAA in tumour samples and anti-tumour activity is presented in Table III. For the purposes of comparison, three additional MAC tumours (MAC 15Aip, MAC 16 and 26) have been included, details of pharmacokinetic exposures at the tumour site have been published elsewhere (Bibby *et al.*, 1987*b*; Bibby *et al.*, 1988; Bibby *et al.*, 1989*a*). There is a poor correlation between pharmacokinetic parameters at the tumour site and the final outcome of chemotherapy *in vivo*. In both the resistant MAC 15A ip and iv tumour lines AUC and peak levels of FAA are higher than these achieved in the very responsive MAC 16 tumour (Table III). Plasma protein binding of FAA is presented in Table IV. There are no significant differences in plasma protein binding between non-tumour and MAC 15A iv and sc tumour bearing mice with values ranging from 68.7% to 85.6%.

The cytotoxic activity of FAA *in vitro* was not enhanced by the inclusion of S-9 or phenobarbitone induced S-9 liver fractions in the incubation mixture (Figure 3). The positive control compound, cyclophosphamide is activated by the S-9 liver preparation. No cell kill was observed in MAC 15A cells exposed to plasma from responding tumour bearing mice.

### Discussion

Two main conclusions can be drawn from the results presented in this study; first, the presence of MAC 15A tumours has a significant influence upon the pharmacokinetic behaviour of FAA and second, that the site-dependent response of MAC 15A tumours cannot be explained on the basis of poor drug bioavailability.

The reasons for altered pharmacokinetics in MAC 15A tumour bearing mice relative to non tumour bearing mice are unknown although it is clearly not due to differences in

**Table I** Plasma and tissue distribution of FAA following the ip administration of FAA (200 mg kg$^{-1}$)

| | $C_{max}$[a] ($\mu g\,g^{-1}$) | $T_{max}$ (h) | $T_{\frac{1}{2}}$ (h) | $K_{el}$ ($h^{-1}$) | $V_d$ ($L\,kg^{-1}$) | $Cl_T$ ($L\,h.^{-1}$) | $AUC_{0-\infty}$[b] ($\mu g\,h.g^{-1}$) |
|---|---|---|---|---|---|---|---|
| *Non tumour bearing mice* | | | | | | | |
| Plasma | 511 | 0.25 | 3.5 | 0.198 | 0.357 | 0.138 | 1443 |
| | 586 | 0.25 | 3.1 | 0.224 | 0.307 | 0.097 | 2058 |
| Kidney | 136 | 0.16 | 2.7 | 0.256 | 1.25 | 0.401 | 499 |
| | 243 | 0.25 | 2.8 | 0.247 | 0.77 | 0.203 | 984 |
| Liver | 239 | 0.25 | 2.7 | 0.256 | 0.661 | 0.277 | 720 |
| | 383 | 0.25 | 3.4 | 0.203 | 0.467 | 0.137 | 1455 |
| Lung | 113 | 0.25 | 1.3 | 0.529 | 1.48 | 0.888 | 225 |
| | 167 | 0.25 | 1.5 | 0.462 | 1.05 | 0.358 | 558 |
| *MAC 15A iv* | | | | | | | |
| Plasma | 520 | 0.25 | 8.6 | 0.080 | 0.37 | 0.056 | 3526 |
| | 474 | 0.25 | 8.5 | 0.081 | 0.39 | 0.041 | 4878 |
| Kidney | 183 | 0.25 | 10.5 | 0.066 | 1.01 | 0.144 | 1384 |
| | 157 | 0.25 | 8.9 | 0.077 | 1.18 | 0.118 | 1697 |
| Liver | 222 | 0.166 | 9.9 | 0.070 | 0.81 | 0.112 | 1770 |
| | 290 | 0.25 | 7.5 | 0.093 | 0.65 | 0.061 | 3237 |
| Lung[c] | 137 | 0.083 | 6.9 | 0.101 | 1.38 | 0.271 | 740 |
| | 119 | 0.25 | 6.4 | 0.107 | 1.54 | 0.287 | 696 |
| *MAC 15A sc* | | | | | | | |
| Plasma | 610 | 0.25 | 9.2 | 0.075 | 0.31 | 0.037 | 5356 |
| | 536 | 0.25 | 8.7 | 0.079 | 0.35 | 0.052 | 3859 |
| Kidney | 155 | 0.25 | 12.6 | 0.055 | 1.14 | 0.112 | 1789 |
| | 239 | 0.25 | 7.3 | 0.094 | 0.82 | 0.098 | 2036 |
| Liver | 375 | 0.08 | 5.8 | 0.119 | 0.51 | 0.072 | 2776 |
| | 361 | 0.25 | 5.4 | 0.129 | 0.53 | 0.078 | 2573 |
| Lung | 169 | 0.25 | 6.9 | 0.101 | 1.11 | 0.198 | 1009 |
| | 192 | 0.25 | 6.9 | 0.101 | 1.01 | 0.162 | 1236 |
| Tumour | 136 | 1 | 12.6 | 0.055 | 0.95 | 0.114 | 1784 |
| | 151 | 1 | 6.9 | 0.101 | 0.83 | 0.159 | 1251 |

[a]Units for plasma levels are μg ml$^{-1}$; [b]Units for plasma AUC values are μg h.ml$^{-1}$; [c]N.B. Lung tissues contain extensive tumour deposits at the time of chemotherapy. For each tissue results from two independent experiments are presented. $K_{el}$ = Elimination constant; $T_{\frac{1}{2}}$ = Terminal half life; $C_{max}$ = Peak concentration ± s.d.; $T_{max}$ = Time of peak concentration; $Cl_T$ = Total body clearance; $V_d$ = Apparent volume of distribution; $AUC_{0-\infty}$ = Area under the curve.

**Table II** Summary of peak plasma and plasma AUC values in tumour bearing and non tumour bearing mice

| | *Peak[a] plasma values* (*μg ml$^{-1}$*) | *Ratio* *peak$_{TB}$[b]* / *peak$_{NTB}$* | *Plasma[a]* *$AUC_{(0-\infty)}$* (*μg h.ml$^{-1}$*) | *Ratio* *$AUC_{TB}$* / *$AUC_{NTB}$* |
|---|---|---|---|---|
| MAC 15A iv | 497 | 0.91 | 4202 | 2.4 |
| MAC 15A sc | 573 | 1.04 | 4608 | 2.6 |
| Non tumour bearing | 549 | 1.00 | 1751 | 1.0 |

[a]Mean values for two experiments with three mice per point in each experiment. [b]TB = Tumour bearing; NTB = Non tumour bearing.

**Table III** Relationship between tumour pharmacokinetic parameters and anti-tumour activity

| | *Dose and route* (*mg kg$^{-1}$*) | *Peak[a] conc* (*μg g$^{-1}$*) | *$AUC_{(0-\infty)}$[b]* (*μg h.g$^{-1}$*) | *% Tumour[c] inhibition* in vivo |
|---|---|---|---|---|
| MAC 15A iv[d] | 200 ip | 128 | 718 | 0 |
| MAC 15A sc | 200 ip | 144 | 1517 | 90 |
| MAC 15A ip[e] | 200 ip | 3100 | 3210 | 0 |
| MAC 16[f] | 200 ip | 41 | 500 | 100 |
| MAC 26[g] | 200 ip | 130 | 1260 | 82 |

[a]Units for peritoneal washings are μg ml$^{-1}$; [b]Units for peritoneal washings are μg h.ml$^{-1}$; [c]Tumour inhibition assessed by: (1) MAC 15A ip and iv: survival times; (2) MAC 15A sc: tumour weight; (3) MAC 16 and 26: growth delay; [d]Peak and AUC values for FAA represent those achieved in lung tissues which contain extensive tumour deposits at the time of chemotherapy; [e]Bibby *et al.*, 1987*b*; [f]Bibby *et al.*, 1988; [g]Bibby *et al.*, 1989*a*; All drug values presented are the mean of two experiments with three mice per point in each experiment.

**Table IV** Plasma protein binding of FAA

| | *Dose* (*mg kg$^{-1}$*) | *Time* (*min*) | *% Binding[a]* *Expt.A.* | *Expt.B.* |
|---|---|---|---|---|
| Non tumour bearing | 200 ip | 15 | 75.8±6.1 | 78.4±4.8 |
| | | 480 | 84.1±5.9 | 81.7±8.2 |
| MAC 15A sc | 200 ip | 15 | 70.8±7.1 | 68.7±7.5 |
| | | 480 | 81.4±3.6 | 85.6±9.1 |
| MAC 15A iv | 200 ip | 15 | 74.2±5.4 | 76.6±8.1 |
| | | 480 | 78.6±4.9 | 85.2±4.7 |

[a]Values presented are the means±standard deviations from two independent experiments (three mice per point in each experiment).

plasma protein binding (Table IV). It also seems unlikely that this phenomenon is a direct result of the dose dependent, non-linear characteristic of FAA pharmacokinetics (Damia *et al.*, 1988; Chabot *et al.*, 1989*b*) as the increase in plasma $AUC_{0-\infty}$ in tumour bearing mice is not accompanied by an increase in peak concentrations (Table II). Whether or not saturation of tubular secretion is involved in this case remains an open question. A much more likely explanation is a combination of host factors such as depression of metabolic capacity, impaired kidney function etc caused by an aggressively growing tumour (Donelli *et al.*, 1984).

Whatever the mechanism, the relevance of these changes to anti-tumour activity appear to be limited, as increases in plasma and tissue $AUC_{0-\infty}$ values for FAA occur in both the resistant MAC 15A iv and the sensitive MAC 15A sc models (Table I). In addition, such significant increases in plasma and tissue AUC values for FAA were not observed in MAC 26 and MAC 16 tumours, both of which are sensitive to FAA (Cummings *et al.*, 1989; Bibby *et al.*, 1988). In terms of tolerance however, these observations may be relevant particularly as Zaharko *et al.* (1986) have shown that increased lethality results from too long an exposure to therapeutically effective concentrations of FAA. Further detailed studies on the relationship between AUC and tolerance in normal and tumour-bearing mice are under way.

The relationship between tumour levels (both peak concentrations and $AUC_{0-\infty}$ values) and anti-tumour activity *in vivo* (Table III) clearly demonstrates that the lack of activity

**Figure 3** *In vitro* chemosensitivity of MAC 15A cells following a 1 h exposure to FAA and cyclophosphamide with and without addition of mouse liver S-9 fractions. Cyclophosphamide (CPA) (◆); FAA (△); CPA + S-9 (◇); FAA + S-9 (▲); FAA + phenobarbitone induced S-9 (○). All points represent the means of three independent experiments±s.d.

against MAC 15A ip and iv tumours is unlikely to be the result of limited drug penetration. Concentrations of FAA in the lungs of unresponsive MAC 15A iv tumour bearing mice ($AUC_{0-\infty}$ = 718 μg h.g$^{-1}$) for example are similar to those achieved in the very responsive MAC 16 tumour (500 μg h.g$^{-1}$). Furthermore, additional studies including Phase I clinical trials have shown that plasma concentrations of FAA associated with activity in mice are achievable in humans which strongly suggests that poor penetration of FAA into human tumours is unlikely to explain the lack of activity observed in clinical studies (Maughan *et al.*, 1989; Kerr *et al.*, 1987).

Two metabolites of FAA were detected *in vivo*. The first metabolite eluted was present at low levels (7% of circulating FAA) in plasma samples but was barely detectable in other tissues. Similar findings have been reported in NMRI mice (Cummings *et al.*, 1989) and Balb/c mice (Damia *et al.*, 1988). The second metabolite could only be detected in liver samples. Attempts to identify cytotoxic metabolites *in vivo* or to activate FAA to a cytotoxic species *in vitro* failed to substantiate the results presented by Chabot *et al.* (1989*a*). In this and previous studies (Damia *et al.*, 1988), no metabolites arising from the inclusion of S-9 liver fractions in the incubation 'mix' *in vitro* were detected by HPLC. Furthermore, as no metabolites could be detected in tumour tissues, their role in the anti-tumour activity of FAA remains questionable.

In conclusion, the results of this study have demonstrated that the site dependent activity of FAA cannot be fully explained on the basis of differences in drug and/or metabolite bioavailability or on altered pharmacokinetic profiles caused by the presence of large tumour loads. The reasons for the site dependent activity of FAA remain unclear although it is likely that the activity of FAA against sc tumours relies in part on the specific vascular feature of tumours at this site.

This research was funded by Bradford's War on Cancer Trust, and Whyte Watson/Turner Cancer Research Trust, Bradford; also Lyonnaise Industrielle Pharmaceutique, Lyon, France. R.M.P. is funded by the Association for International Cancer Research.

### References

BIBBY, M.C., DOUBLE, J.A., ALI, S.A., FEARON, K.C.H., BRENNAN, R.A. & TISDALE, M.J. (1987*a*). Characterisation of a transplantable adenocarcinoma of the mouse colon producing cachexia in recipient animals. *J. Natl Cancer Inst.*, **78,** No. 3. 539.

BIBBY, M.C., DOUBLE, J.A., PHILLIPS, R.M. & LOADMAN, P.M. (1987*B*). Factors involved in the anti-cancer activity of the investigational agents LM985 (flavone acetic acid ester) and LM975 (flavone acetic acid). *Br. J. Cancer*, **55,** 159.

BIBBY, M.C., DOUBLE, J.A. & LOADMAN, P.M. (1988). Unique chemosensitivity of MAC 16 tumours to flavone acetic acid (LM975, NSC 347512). *Br. J. Cancer*, **58,** 341.

BIBBY, M.C., DOUBLE, J.A., LOADMAN, P.M. & DUKE, C. (1989*a*). Reduction of tumour blood flow by flavone acetic acid: a possible component of therapy. *J. Natl Cancer Inst.*, **81,** 216.

BIBBY, M.C., PHILLIPS, R.M. & DOUBLE, J.A. (1989*b*) Influence of site on the chemosensitivity of transplantable murine tumours to flavone acetic acid (LM975, NSC 347512). *Cancer Chemother. Pharmacol.*, **24,** 87.

CHABOT, G.G., BISSERY, M.C. & GOUYETTE, A. (1989*a*). Flavone acetic acid (LM975 NSC347512) activation to cytotoxic species *in vivo* and *in vitro*. *Cancer Chemother. Pharmacol.*, **24,** 273.

CHABOT, G.G., BISSERY, M.C., CORBETT, T.H., RUTKOWSKI, K. & BAKER, L.H. (1989*b*). Pharmacodynamics and causes of dose dependent pharmacokinetics of flavone 8 acetic acid (LM975; NSC-347512) in mice. *Cancer Chemother. Pharmacol.*, **24,** 15.

CORBETT, T.H., BISSERY, M.C., WOZNIAK, A. & 5 others (1986). Activity of flavone acetic acid (NSC-347512) against solid tumours of mice. *Invest. New Drugs*, **4,** 207.

CUMMINGS, J., DOUBLE, J.A., BIBBY, M.C. & 5 others (1989). Characterisation of the major metabolites of flavone acetic acid and comparison of their disposition in man and mouse. *Cancer Res.*, **49,** 3587.

DAMIA, G., ZANETTE, M.L., ROSSI, C., MANDELLI, R., FERRARI, A. & D'INCALCI, M. (1988). Dose dependent pharmacokinetics of flavone acetic acid in mice. *Cancer Chemother. Pharmacol.*, **22,** 47.

DONELLI, M.G., D'INCALCI, M. & GARATTINI, S. (1984). Pharmacokinetic studies of anti-cancer drugs in tumour bearing animals. *Cancer Treat. Rep.*, **68,** 381.

DOUBLE, J.A., BALL, C.R. & COWEN, P.N. (1975). Transplanation of adenocarcinoma of the colon in mice. *J. Natl Cancer Inst.*, **54,** 271.

DOUBLE, J.A., BIBBY, M.C. & LOADMAN, P.M. (1986). Pharmacokinetics and anti-tumour activity of LM985 in mice bearing transplantable adenocarcinoma of the colon. *Br. J. Cancer*, **54,** 595.

GIAVAZZI, R., GAROFALO, A., DAMIA, G., GARATTINI, S. & D'INCALCI, M. (1988). Response to flavone acetic acid (NSC 347512) of primary and metastatic human colorectal carcinoma xenografts. *Br. J. Cancer*, **57,** 277.

GIBALDI, M. (1984). Introduction to pharmacokinetics. In *Biopharmaceutics and Clinical Pharmacokinetics* (third edition) p.1. Gibaldi, M. (ed.). Lea and Febiger: Philadelphia.

KERR, D.J., KAYE, S.B., CASSIDY, J. & 6 others (1985). A clinical pharmacokinetic study of LM 985 and LM 975. *Br. J. Cancer*, **52,** 467.

KERR, D.J., KAYE, S.B., CASSIDY, J. & 7 others (1987). Phase I and pharmacokinetic study of flavone acetic acid. *Cancer Res.*, **47,** 6776.

KERR, D.J., MAUGHAN, T., NEWLANDS, E. & 4 others (1989). Phase II trials of flavone acetic acid in advance malignant melanoma and colorectal cancer. *Br. J. Cancer*, **60,** 104.

MAUGHAN, T.S., WARD, R., WORKMAN, P. & BLEEHEN, N. (1989). Tumour concentration of flavone acetic acid (FAA) in human melanoma. *Proc. 5th European Conference on Clinical Oncology*, London. (Abst. No. 0-0131).

PHILLIPS, R.M., BIBBY, M.C. & DOUBLE, J.A. (1988). Experimental corrections of *in vitro* drug sensitivity with *in vivo* responses to ThioTEPA in a panel of murine colon tumours. *Cancer Chemother. Pharmacol.*, **21,** 165.

PLOWMAN, J., NARAYANAN, V.L., DYKES, D. & 4 others (1986). Flavone acetic acid: a novel agent with preclinical antitumour activity against colon adenocarcinoma 38 in mice. *Cancer Treat. Rep.*, **70,** 631.

ZAHARKO, D.S., GRIESHABER, C.K., PLOWMAN, J. & CRADOCK, J.C. (1986). Therapeutic and pharmacokinetic relationships to flavone acetic acid: an agent with activity against solid tumours. *Cancer Treat. Rep.*, **70,** 1415.

ZWI, J.L., BAGULEY, B.C., GAVIN, J.B. & WILSON, W.R. (1989). Blood flow failure as a major determinant in the anti-tumour activity of flavone acetic acid. *J. Natl Cancer Inst.*, **81,** 1005.